# Time-Calibrated Phylogenomics of the Classical Swine Fever Viruses: Genome-Wide Bayesian Coalescent Approach

**DOI:** 10.1371/journal.pone.0121578

**Published:** 2015-03-27

**Authors:** Taehyung Kwon, Sook Hee Yoon, Kyu-Won Kim, Kelsey Caetano-Anolles, Seoae Cho, Heebal Kim

**Affiliations:** 1 Department of Agricultural Biotechnology and Research Institute for Agriculture and Life Sciences, Seoul National University, Seoul, 151-921, Republic of Korea; 2 Interdisciplinary Program in Bioinformatics, Seoul National University, Seoul, 151-747, Republic of Korea; 3 Department of Animal Sciences, University of Illinois, Urbana, IL, 61801, United States of America; 4 C&K Genomics Inc. 514 Main Bldg., Seoul National University Research Park, San 4-2 Boncheon-dong, Gwanak-gu, Seoul, 151-919, Republic of Korea; Sun Yat-sen University, CHINA

## Abstract

The phylogeny of classical swine fever virus (CSFV), the causative agent of classical swine fever (CSF), has been investigated extensively. However, no evolutionary research has been performed using the whole CSFV genome. In this study, we used 37 published genome sequences to investigate the time-calibrated phylogenomics of CSFV. In phylogenomic trees based on Bayesian inference (BI) and Maximum likelihood (ML), the 37 isolates were categorized into five genetic types (1.1, 1.2, 2.1, 2.3, and 3.4). Subgenotype 1.1 is divided into 3 groups and 1 unclassified isolate, 2.1 into 4 groups, 2.3 into 2 groups and 1 unclassified isolate, and subgenotype 1.2 and 3.4 consisted of one isolate each. We did not observe an apparent temporal or geographical relationship between isolates. Of the 14 genomic regions, NS4B showed the most powerful phylogenetic signal. Results of this evolutionary study using Bayesian coalescent approach indicate that CSFV has evolved at a rate of 13×.010^-4^ substitutions per site per year. The most recent common ancestor of CSFV appeared 2770.2 years ago, which was about 8000 years after pig domestication. The effective population size of CSFV underwent a slow increase until the 1950s, after which it has remained constant.

## Introduction

Classical swine fever (CSF), also known as hog cholera is a highly contagious viral disease of domestic pig and wild boar that causes watery diarrhea and weakness. The high mortality rate of CSF leads to significant economic losses in the global swine industry [1]. From the 1990s to the early 2000s, sporadic outbreaks of CSF in European swine industries were reported [2–4]; for instance, 117, 48, and 429 farms were contaminated in Germany, Belgium, and the Netherlands respectively. In the case of the Netherlands, economic losses were calculated to be almost 2.3 billion US dollars [5, 6]. Many countries are currently considered to be CSF free or low risk by the United States Department of Agriculture (USDA), including Australia, Canada, and several European countries. Given the potential for devastating economic impact of a CSF outbreak on the swine industry, circumspect governmental regulations are in place to prevent CSF exposure in large, dense populations of pigs. Nevertheless, CSF outbreaks in small populations of wild boar are still observed in several regions like India [7].

As populations in the swine industry become denser and larger, more government attention to CSF is needed. Additionally, countries that are not CSF free, such as India or China, account for a large portion of the global swine industry [8], which means that global attention to the CSF is necessary as well.

Classical swine fever virus (CSFV), the causative agent of CSF, is a member of the genus *Pestivirus* within the family *Flaviviridae* [1, 9]. In viral epidemiology, mortality rate is defined as the number of deaths over the number of individuals infected during a specific period. [10]. While the mortality rate of CSF is high, the severity differs by case, especially depending on the age of the animal and virulence of the virus. While symptoms in older animal tend to be milder, the mortality rate of young pigs is almost 90 percent [1, 9]. CSF can often be confused with American swine fever (ASF), which produces clinical symptoms similar in pigs to that of CSF, including haemorrhagic fever. However, despite these similarities, CSF virus is a small single-stranded RNA virus, while ASF virus is a large double-stranded DNA virus of genus *Asfivirus* in the family *Asfarviridae* [11]. While it is difficult to diagnose these viruses from clinical signs and lesions alone, confirmatory experiments like RT-PCR is necessary [12]. CSFV is also structurally close to the bovine viral diarrhea (BVD) virus and the border disease (BD) virus, which also belong to the genus *Pestivirus* [9]. Although these viruses are capable of infecting pigs, they do not have the ability to spread out without their original hosts and can easily be genetically differentiated from CSFV [13].

CSFV has a single positive-stranded RNA genome of approximately 12.3 kb including 11.7 kb open reading frame (ORF), which is flanked by the 5’ and 3’untranslated regions (UTRs). The ORF encodes 12 polypeptides, which contain four structural proteins (C, E^rns^, E1, E2) and seven non-structural proteins (p7, NS2, NS3, NS4A, NS4B, NS5A, NS5B) with autoprotease (N^pro^) at N-terminus [14, 15]. The UTRs contain signals for viral replication, transcription, and translation [15]. Ji et al. [16] found that virulence of CSFV is associated with mutations in seven protein genes (Npro, C, Erns, E1, E2, p7, NS4B) related to viral entry, release, replication and host cell interaction [17].

CSFVs have been classified under three major genotypes (1, 2, and 3), each comprised of three to four subgenotypes (1.1–1.4; 2.1–2.3; 3.1–3.4) [18, 19]. Since the first report of CSFV in the United States at 1833 [15] and in South Korea at 1908 [20], many global phylogenetic studies have been published based on the genome [21–23] and particular genes such as 5’ UTR, E2, and NS5B (e.g., [24–31]). However, as described in Floegel et al. and Ji et al. [32, 33], accumulation of mutations in viruses have caused variability in virulence between genotypes. The temporal dynamics of global CSFVs also remain unclear. The only previous study analyzed a small number genes (5’UTR, N^pro^, and E2) [34], and found that the most recent common ancestor of the virus existed in the year 1892.

To fully comprehend the phylogeny and evolutionary dynamics of CSFV, as mentioned in previous phylogenomic studies [35], it is necessary to conduct a genomic scale analysis. Here, we performed genome wide study on the time-calibrated phylogenomics of the virus. We analyzed 37 available public genome sequences that sampled during a time period of 68 years (1945–2012). The objectives of this study are (1) to analyze the characteristics of CSFV genome sequences; (2) to reconstruct the genome wide phylogeny of the global CSFVs using two different analyses methods, Bayesian inference (BI) and maximum likelihood (ML); and (3) to elucidate the evolutionary mechanisms such as selection pressure, substitution rate, divergence times, and effective population size changes using Bayesian coalescent approach.

## Materials and Methods

### Sequence and selection pressure analyses

We used the published nucleotide and amino acid sequences of 37 CSFV isolates from NCBI, discovered around the world from 1945 to 2012. These isolates consisted of 20 Asian isolates (China, 14; South Korea, 2; Taiwan, 3; Japan, 1), 16 European isolates (Germany, 7; France, 3; Italy, 1; Lithuania, 1; Spain, 1; Denmark, 1; Bulgaria, 1; Croatia, 1), and 1 American isolate (USA, 1). Each isolate belongs to one of the following subgenotypes: 1.1, 1.2, 2.1, 2.3, and 3.4. The information of 37 samples is shown in Table 1.

**Table 1:** Information of the isolates used in this study.

Isolate	Subgenotype	Country	Year	Accession no.
CSFV-GZ-2009	1.1	China	2009	HQ380231
CF114	1.1	China	2001	AF333000
Shimen	1.1	China	1945	AF092448
SWH	1.1	China	2004	DQ127910
C-ZJ-2008	1.1	China	2008	HM175885
JL1(06)	1.1	China	2006	EU497410
LOM	1.1	Japan	1980	EU789580
Eystrup	1.1	Germany	1964	NC_002657
Alfort/187	1.1	France	1968	X87939
CAP	1.1	France	1978	X96550
Brescia	1.1	Italy	1945	AF091661
Glentorf	1.1	Denmark	1968	U45478
BRESCIAX	1.2	USA	2001	AY578687
YC11WB	2.1	South Korea	2011	KC149990
PC11WB	2.1	South Korea	2011	KC149991
HNLY-2011	2.1	China	2011	JX262391
Heb52010	2.1	China	2010	JQ268754
HNSD-2012	2.1	China	2012	JX218094
SXCDK	2.1	China	2009	GQ923951
SXYL2006	2.1	China	2006	GQ122383
GXWZ02	2.1	China	2002	AY367767
HEBZ	2.1	China	2009	GU592790
Zj0801	2.1	China	2008	FJ529205
96TD	2.1	Taiwan	1996	AY554397
0406/CH/01/TWN	2.1	Taiwan	2001	AY568569
Paderborn	2.1	Germany	1997	GQ902941
CSFV/2.1/dp/CSF1048/2009/LT/Penevezys	2.1	Lithuania	2009	HQ148063
CSFV/2.3/wb/XXX0609/2004/Uelzen	2.3	Germany	2004	GU324242
CSFV/2.3/wb/CSF1045/2009/Roesrath	2.3	Germany	2009	GU233734
CSFV/2.3/wb/XXX0608/2005/Euskirchen	2.3	Germany	2005	GU233732
CSFV/2.3/wb/CSF1046/2009/Hennef	2.3	Germany	2009	GU233733
CSFV/2.3/dp/CSF857/2006/Borken	2.3	Germany	2006	GU233731
Alfort/T	2.3	France	1980	J04358
Sp01	2.3	Spain	2001	FJ265020
CSFV/2.3/dp/CSF864/2007/BG/Jambul	2.3	Bulgaria	2007	HQ148062
CSFV/2.3/dp/CSF0821/2002/HR/Novska	2.3	Croatia	2002	HQ148061
94.4/IL/94/TWN	3.4	Taiwan	1994	AY646427

Both nucleotide and amino acid sequences of the CSFV whole genome and 14 regions (5’UTR-Npro-C-Erns-E1-E2-p7-NS2-NS3-NS4A-NS4B-NS5A-NS5B-3’UTR) were aligned with MAFFT 7 [36]. The following values were calculated from aligned sequences using Tree puzzle 5.2 [37] and BIOEDIT 7.2.3.0 [37, 38]: total sites (including gaps), conserved sites, and average identities. During the process, we depicted variable site plots for nucleotide and amino acid sequence. Results of model tests using Modeltest 3.7 [39] for the genome and genomic regions are summarized in Table 2.

**Table 2:** The best fit evolutionary models estimated for CSFV genomic regions with Modeltest 3.7.

Genomic region	Model	-lnL	Base frequencies (A,C,G)	Substitution matrix (A-C, A-G, A-T, C-G, C-T)	Pinvar	Nst
Whole genome	GTR+I+G	66705.17	0.31, 0.22, 0.26	0.99, 11.00, 1.90, 0.38, 25.04	0.49	6
5'UTR	SYM+I+G	1451.45	Equal	0.96, 3.90, 1.20, 0.00, 9.47	0.53	6
N^pro^	GTR+G	2755.61	0.30, 0.23, 0.27	3.67, 26.01, 3.40, 0.72, 62.76	0.00	6
C	GTR+I	1595.19	0.38, 0.18, 0.27	1.42, 9.28, 1.63, 0.16, 26.64	0.55	6
E^rns^	GTR+I+G	3562.38	0.32, 0.22, 0.27	1.90, 13.50, 2.24, 0.46, 38.94	0.45	6
E1	GTR+I	3163.49	0.29, 0.23, 0.25	1.12, 17.99, 2.95, 0.56, 27.72	0.56	6
E2	GTR+I+G	6623.00	0.28, 0.22, 0.27	1.01, 9.54, 1.59, 0.33, 19.16	0.50	6
p7	TrN+G	1072.38	0.30, 0.19, 0.23	1.00, 12.65, 1.00, 1.00, 22.66	0.00	6
NS2	GTR+I+G	7931.16	0.30, 0.21, 0.25	0.85, 9.80, 1.42, 0.29, 18.59	0.42	6
NS3	GTR+I+G	9710.91	0.31, 0.22, 0.27	0.91, 12.27, 2.36, 0.34, 29.50	0.59	6
NS4A	TIM+I	947.92	0.34, 0.21, 0.23	1.00, 57.99, 5.13, 5.13, 111.19	0.55	6
NS4B	GTR+I+G	5770.45	0.30, 0.22, 0.27	1.23, 13.30, 2.18, 0.29, 28.53	0.54	6
NS5A	GTR+I+G	8511.48	0.32, 0.22, 0.26	0.90, 9.71, 1.71, 0.41, 21.26	0.42	6
NS5B	GTR+I+G	11343.28	0.33, 0.21, 0.26	0.96, 11.95, 2.35, 0.57, 35.59	0.49	6
3'UTR	TVM+G	1190.08	0.31, 0.21, 0.16	0.24, 5.22, 1.04, 0.60, 5.22	0.00	6

To evaluate selective pressure acting on CSFVs, the relative rates of nonsynonymous and synonymous substitution (ω = dN/dS) across coding region of the viral genome were also estimated using ClustalX 1.81 [40] PAL2NAL [41], and CodeML of PAML 4.7 package [42]. A dN/dS ratio of < 1 indicated purifying selection, dN/dS = 1 suggested an absence of selection (i.e., neutral evolution), and dN/dS > 1 indicated positive selection.

### Phylogenomic tree reconstruction

Phylogenomic trees were reconstructed using two different analytical methods—BI and ML. The best-fit model for the CSFV whole genome was determined using Akaike’s information criterion (AIC) within Modeltest 3.7 [39]. BI analysis using MrBayes 3.1.2 [43] was performed with following options: nst = 6, rates = invgamma, number of generation = 20,000,000, and burn-in = 20,000. Bayesian posterior probability (BPP) values were shown on the BI tree [44]. ML analysis using Phyml 3.0 [45] was also conducted with the following parameters: model of nucleotide substitution = GTR, replicates = 500, pinvar = estimated, and number of substitution rate categories = 6. All trees were visualized in Figtree 1.4 [46].

In order to screen for congruent tree topologies with genome tree topologies, all 14 genomic regions were analyzed in the same methods as genome data. The best-fit models for each region used in analysis are summarized in Table 2.

### Estimation of substitution rate, divergence times, and population size changes

Using BEAST 1.7.4 [47], the mean rate of nucleotide substitution, time of the most recent common ancestor (tMRCA), and change in effective population size of the CSFV were estimated with the result of the model test. We combined three molecular clock models (strict, relaxed uncorrelated exponential, and relaxed uncorrelated log-normal) with five demographic models (constant size, exponential growth, expansion growth, logistic growth, and Bayesian skyline) to make 15 datasets for simulation. Next, each dataset was simulated with the following options: generation = 400,000,000, burn-in of 10%, and ESSs > 100. By comparing the highest Bayesian factors (log_10_ Bayesian factor > 2) which were based on the relative marginal likelihood of 15 models, the relaxed uncorrelated exponential clock and expansion growth population model was selected as the best-fit evolutionary model. The resulting convergence was analyzed using Tracer 1.5 [48]. Trees were summarized as maximum clade credibility (MCC) tree using the TreeAnnotator 1.7.4 [49] and visualized using Figtree 1.4 [46]. For each tree node, estimated divergence times and 95% highest posterior density (HPD) intervals, which summarize the statistical uncertainties, were indicated. The change of effective population size was plotted using Bayesian skyline plot (BSP) analyses [50].

## Results

### Sequence and selection pressure analyses

**Fig 1:**
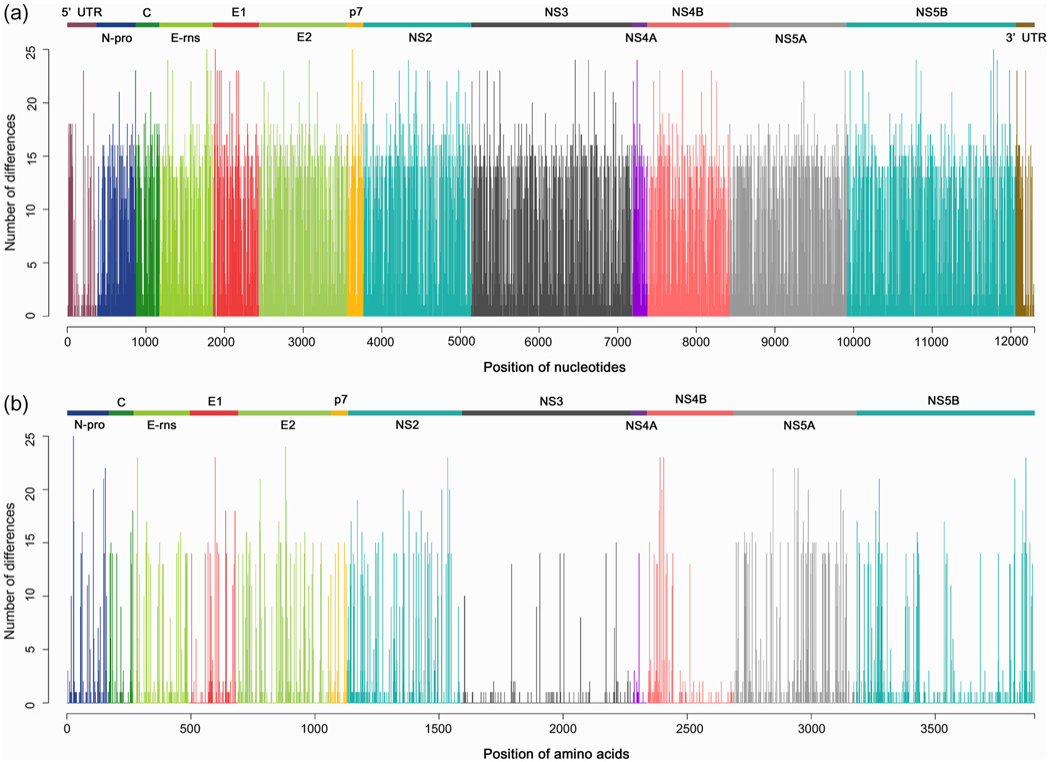
Variable site plots for nucleotide (a) and amino acid (b) sequence of 37 CSFVs. The number of differences at each site represents the number of variable isolates estimated with multiple sequence alignment. Each color indicates a different genomic region.

Features of the entire genome and 14 individual regions of the 37 CSFVs are summarized in Table 3. The whole genome alignment (including insertions) was 12,301 bps in length, and revealed relatively low similarities; 7,645 (62.1%) of the nucleotide sites were conserved. The differences in nucleotide and amino acid sequence at each site of the CSFV genome alignment are shown in Fig. 1. Both nucleotide and amino acid variations were evenly distributed throughout the genomes, though higher amino acid similarities were observed in three regions (NS3, NS4A, and NS4B). Pairwise comparisons also revealed that the average identities among the complete genome sequences were 89.3% for nucleotide sequences. Of the 14 individual regions, E2 was the most variable (average sequence identities of 87.8 and 92.3% for nucleotides and amino acids, respectively), while 5’ UTR was the most conserved (average sequence identities of 94.2% for nucleotides).

**Table 3:** Summary of alignment of CSFV genomic regions.

Genomic region	Total sites including gaps, nt/aa	Conserved sites(%), nt/aa	Average identities(%), nt/aa	ω value (dN/dS)
Whole genome	12301/NA	7645(62.1%)/NA	89.3/NA	NA
ORF	11694/3898	7224(61.8%)/2949(75.7%)	89.1/94.7	0.067
5'UTR	374/NA	287(76.7%)/NA	94.2/NA	NA
N^pro^	504/168	301(59.7%)/117(69.6%)	89.6/93.2	0.137
C	297/99	183(61.6%)/69(69.7%)	88.7/93.7	0.093
E^rns^	681/227	423(62.1%)/167(73.6%)	89.4/93.7	0.081
E1	585/195	360(61.5%)/153(78.5%)	88.7/95.3	0.069
E2	1119/373	654(58.4%)/261(70.0%)	87.8/92.3	0.104
p7	210/70	126(60.0%)/51(72.9%)	88.1/94.2	0.071
NS2	1371/457	803(58.6%)/318(69.6%)	88.0/92.7	0.089
NS3	2049/683	1400(68.3%)/609(89.2%)	90.7/98.6	0.020
NS4A	192/64	120(62.5%)/55(85.9%)	89.6/98.3	0.032
NS4B	1041/347	653(62.7%)/280(80.7%)	89.3/96.6	0.049
NS5A	1491/497	858(57.5%)/332(66.8%)	88.6/92.1	0.094
NS5B	2154/718	1343(62.3%)/537(74.8%)	89.4/95.0	0.065
3'UTR	230/NA	133(57.8%)/NA	89.2/NA	NA

**Fig 2:**
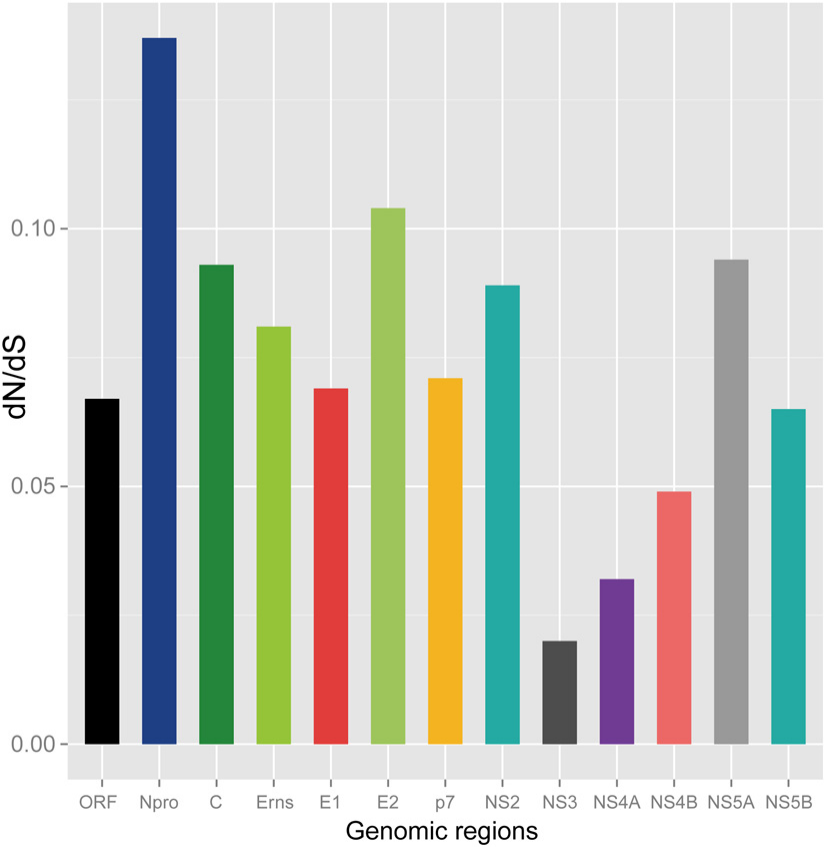
Pairwise dN/dS (ω) values of the protein coding gene sequences of 37 CSFVs.

The mean ratio of nonsynonymous/synonymous substitution (dN/dS) derived from entire data sets was calculated and indicated that purifying selection acted on the CSFV genome sequences (Table 3, Fig. 2). The dN/dS value of entire coding sequences was 0.067 and all values for each component gene were lower than 1. Particularly, the highest dN/dS ratio was observed in N^pro^ (0.137), while the lowest one was shown in NS3 (0.020).

### Phylogenomic tree reconstruction

**Fig 3:**
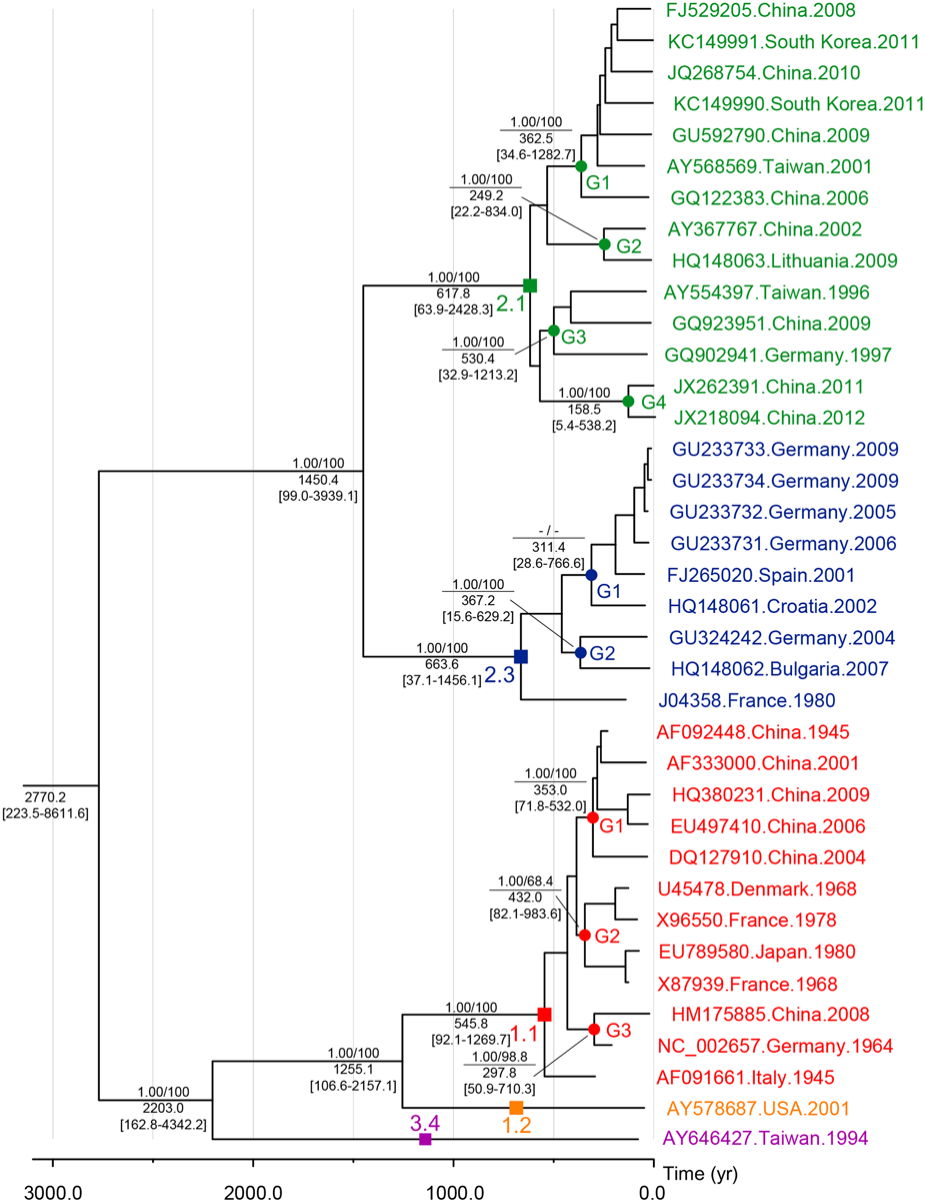
Bayesian maximum clade credibility phylogenetic tree based on the whole genome sequences of 37 CSFVs. With the BI and ML methods, identical topology was produced. Divergence times (in years) are positioned below the nodes; the 95% HPD intervals are indicated in brackets. The confidence of the phylogenetic analysis is presented above the nodes: left numbers represent Bayesian posterior probabilities (≥ 0.80) and right ones represent ML bootstrap values (≥ 60%). Subgenotypes and groups are indicated above the corresponding nodes using squares and circles.

37 CSFV isolates were classified into one of five subgenotypes (1.1, 1.2, 2.1, 2.3, and 3.4). BI and ML methods presented similar configurations regarding the phylogenomics of CSFV, and also supported the topology of the maximum clade credibility (MCC) tree (Fig. 3). The phylogenomic tree revealed two sister-group relationships, subgenotype 1.1 + 1.2 + 3.4 and subgenotype 2.1 + 2.3, with a branching pattern of [{3.4, (1.1, 1.2)}, (2.1, 2.3)]. Subgenotype 1.1 consisted of 11 CSFVs collected from Asia (China and Japan) and Europe (Denmark, France, Germany, and Italy) during 1945–2009; those 11 viruses were divided into 3 groups and 1 unclassified isolate. Subgenotype 2.1 was grouped with 9 isolates from Asia (South Korea, China, and Taiwan) and Europe (Germany and Lithuania) during 1996–2012; individuals were clustered to 4 groups. On the other hand, all viruses in subgenotype 2.3 originated from European countries (France, Germany, Spain, Croatia, and Bulgaria) between 1980 and 2009; individuals were categorized to 2 groups and 1 unclassified isolate. Subgenotype 1.2 and 3.4 had only one member derived from USA in 2001 and Taiwan in 1994.

**Fig 4:**
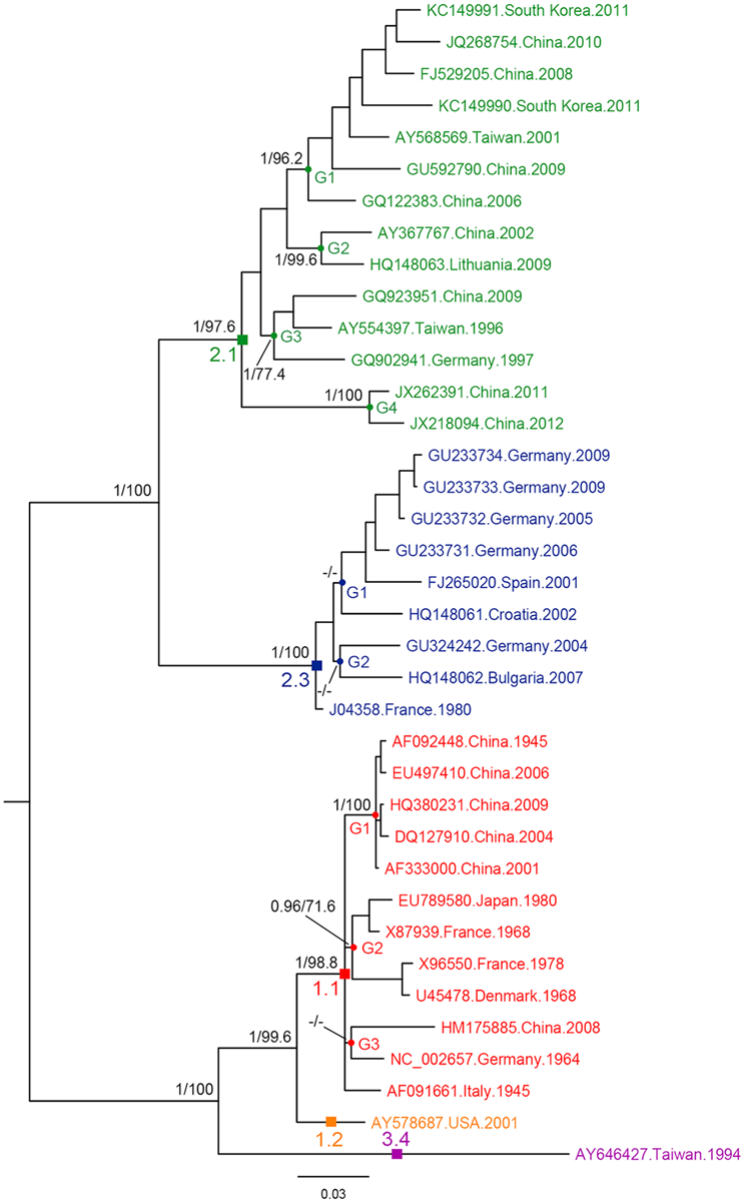
The BI tree based on NS4B sequences of 37 CSFVs. The robustness of the phylogenetic analysis is showed above the nodes: left numbers are Bayesian posterior probabilities (≥0.80) and right ones are ML bootstrap values (≥60%). Subgenotypes and groups are marked above the corresponding nodes using squares and circles.

To identify the most significant phylogenetic marker of CSFV among the 14 regions, phylogenetic trees for the 14 regions were reconstructed based on both BI and ML methods, and these individual trees were compared to the whole genome trees. NS4B gene trees were the most similar to the genomic tree, thus, NS4B gene was chosen as the most significant phylogenetic marker for CSFV (Fig. 4). The overall tree topologies of E2 gene, which was used as the marker of CSFV phylogeny, were very different from those of genome tree (S1 and S2 Figs.); most groupings were disrupted in the E2 trees.

### Substitution rate, divergence times, and population size changes

**Fig 5:**
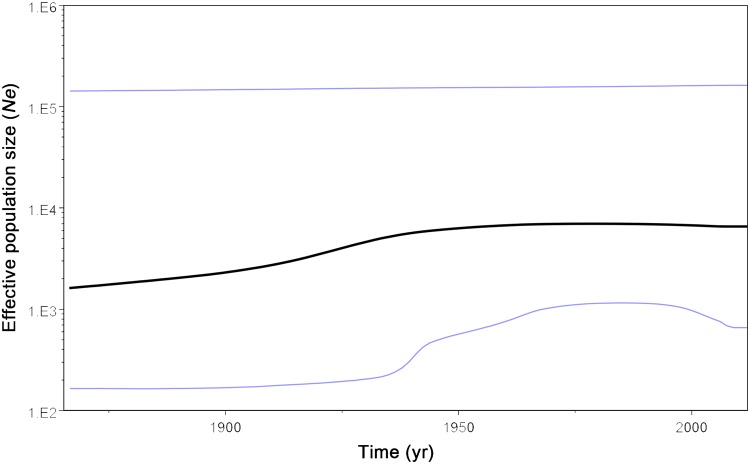
Bayesian skyline plot based on the entire genome sequences of 37 CSFV isolate. The plot depicts changes in the effective population size (*N*_*e*_). The dark line shows the effective population size estimated through time. The upper and lower lines indicate the 95% HPD range of BSP.

Results of analyses performed by BEAST 1.7.4 revealed that the relaxed uncorrelated exponential clock and expansion growth population model was the best-fit for simulating evolution of CSFV genome. Under this model, evolutionary rate was estimated as 1.03×10^-4^ substitution/site/year, and the tMRCA of 37 CSFVs was estimated as 2770.2 years ago (95% HPD 223.5–8611.6). According to the MCC tree, subgenotype 3.4 appeared first 2203.0 (95% HPD = 612.8–4342.1) years ago, followed by sequential divergence of subgenotype 1.2 (1255.1 years ago; 95% HPD = 106.6–2157.1), 2.3 (663.6 years ago; 95% HPD = 37.1–1456.1), 2.1 (617.8 years ago; 95% HPD = 63.9–2428.4), and 1.1 (545.8 years ago; 95% HPD = 92.1–1269.7) (Fig. 3). In BSP, the effective population size of CSFV slowly increased until the 1950s, after which it plateaued (Fig. 5).

## Discussion

Our research aimed to characterize the CSFV genome and elucidate its evolutionary features using genomic data. Our results revealed a high degree of genetic diversity between the 37 CSFV genome sequences. This feature confirmed the views of Domingo et al. [51] who proposed that RNA viruses have the high level of mutation rates of 10^-3^ to 10^-5^ nucleotide substitutions per site per replication cycle due to their inaccurate RNA replication. Of the 14 genomic regions, our analyses showed that E2 was significantly (p-value < 2.2 × 10^-16^) variable, while 5’UTR was the most conserved. The E2 protein, a glycoprotein formed on the virus membrane, has been suggested as a virulence determinant [52, 53]. In CSFV, it acts as an immunogenic protein which induces the neutralizing antibodies and protection reactions of the host [54]. 5’UTR of CSFV contains an internal ribosome entry site (IRES) where translation initiation occurs, which is indispensable for the virus [55, 56]. Domains in IRES tend to be highly conserved between several viruses in genus *Pestivirus*, including CSFV [55, 57]. These two regions (E2 and 5’UTR) have been considered as the most important targets for CSFV studies such as PCR detection and immunology as well as evolutionary phylogenetics. Because the sequence dissimilarity may affect PCR diagnosis and vaccination efficiency as well as investigations on immunology, epidemiology, and phylogenetic evolution, it is necessary to continuously monitor the genome sequence variability.

In agreement with the interpretations of Ji et al. [33], our results on the basis of complete coding genome sequences were also indicative of purifying selection acting on the CSF viruses; the mean ratio of nonsynonymous/synonymous substitution (ω = dN/dS) values for each genomic region were low in all cases (all, ω < 1). Of those, the N^pro^ presented the highest dN/dS values, while the NS3 indicated the lowest ones.

Next, the phylogeny of CSFV was studied to provide further information regarding epidemiology and evolution. Despite of the importance of CSFV to the pig industry and recent rapid increase of CSFV data, most phylogenetic studies describing the virus have focused only on limited genome region sequences such as E2 [29, 30, 58, 59], 5’UTR [25, 26, 29, 30], and NS5B [31, 60]. However, expanding information of genome-wide phylogeny has potential to improve our knowledge on its emergence and transmission. Our phylogenomic analysis of 37 CSFV genome sequences revealed that no apparent correlation between time and/or country and the evolution of the virus. Within the subgenotype 1.1, the isolates appeared in samples taken in four European countries (Denmark, France, Germany, and Italy) and two Asian countries (China and Japan) during 1945–2009. Subgenotype 2.1 viruses originated from six countries (South Korea, China, Taiwan, Germany, and Lithuania) during 1996–2012. Moreover, isolates of subgenotype 2.3 were found in five countries (France, Germany, Spain, Croatia, and Bulgaria) during 1980–2009. These configurations were also in concordance with the views of other authors [23, 35] and may be largely due to their rapid spread via the frequent international trade in livestock. The mixed population structure can make vaccine development and local regulation more difficult. Thus, it is essential to continuously monitor the structural changes of the mixed population.

In order to trace the most appropriate phylogenetic marker, we compared 14 individual gene trees with complete genome trees. Although E2, NS5B, and 5’UTR are generally considered suitable for elucidating the CSFV phylogeny, our findings postulated that topologies of NS4B trees were the most similar to those of the complete genome trees, rather than E2, NS5B, and 5’UTR. Thus, we suggest that NS4B has the strongest signal to infer the genetic relationships of these viruses. Among three frequently used markers, topologies of E2 tree was more apparent than those of the other two regions. Given that research has shown that the complete E2 sequence is suitable for phylogenetic analysis of CSFV [61], comparison of topologies between NS4B and E2 full-length sequences can support the discriminatory power of NS4B as a phylogenetic marker (S1 and S2 Figs.). Both of those regions are regarded as significant determinant in viral activity. That is, E2 is related to viral entry to the target cell [62], E2 has been known as a virulence determinant of CSFV, and as the most immunogenic factor among the viral protein [63, 64]. The nonstructural protein translated from NS4B was reported to contain Toll/Interleukin-1 receptor (TIR) domain in Brescia strain which is considerably virulent, and the viral replication of Brescia strain was shown to be decreased by mutation in that domain [65]. Additionally, after artificial re-injection of the GPE- vaccine, amino acid sequence changes in NS4B contributed to the recovery of virulence of the virus [66]. Thus, NS4B was considered to be essential in viral replication and to have interaction with immune system, and mutations in NS4B significantly affected the virulence of CSFV [65].

Finally, we attempted to elucidate the evolutionary mechanisms and features of CSFV including estimation of evolutionary rate, divergence times, and population size changes. Regarding the evolutionary dynamics of CSFV, there was only one previous study [34]. They utilized only three genes (5’UTR, N^pro^, and E2) sequences of 35 pestiviruses including six CSFVs, and reported that the most recent common ancestor of CSFV existed 1825 years ago; no analysis of substitution rate and population size changes was performed. In contrast to this small number of genes analyzed from a limited selection of representative viruses, we analyzed the complete genome sequences with an available year of isolation in order to co-estimate an overall substitution rate, population size changes as well as divergence times for CSFVs.

The mean evolutionary rate estimated in the present study was 1.03×10^-4^ (95% HPD 2.03×–2.61×10^-4^) substitutions/site/year; that calculation was within the range of 10^-2^ to 10^-5^ nucleotide substitutions/site/year for nearly all RNA viruses [67]. However, this value was lower than the results from previous studies based on E2 sequences; the mean evolutionary rates were calculated as 3.3×10^-3^ and 2.41×10^-3^ substitutions/site/year in two previous studies [18, 62]. Because E2 was relatively variable and less conserved compared to the whole genome, the mean rate of substitution of the whole genome could be lower than that of E2. The fast evolutionary rates of RNA viruses including CSFV are affected by a combination of forces such as lack of proof-reading, small genome size, short generation times, and the extent of natural selection [68]. As a result, it is possible that RNA virus raises viral population adaptation, survival, and fitness, allowing them to rapidly spread to new hosts and novel environments [69]. The tMRCA of CSFV was 2770.2 (95% HPD 223.5–8611.6) years ago which was about 8000 years later than the domestication of wild boar [70, 71].

In terms of the effective population size changes of CSFVs, our bayesian skyline plot (BSP) analysis depicted that a population increase until the middle of 1990s, after which population size appears to have evolved at a near constant rate population size until the present. The trend of effective population size can be attributed to the introduction of vaccine to prevent further global CSF outbreaks [72]. This plateau after 1950s also can be explained by huge slaughter of pigs during global outbreak of porcine reproductive and respiratory syndrome (PRRS) and swine influenza (SI) [73–75].

The present study is the first of its kind to use the complete CSFV genome to investigate the temporal dynamics of the virus. CSFV is still one of the most acute pathogens in the global swine industry. Accordingly, global strategies are essential for prevention and control of this virus. Results of the present study expand the limited information available on CSFV evolutionary dynamics, which may be crucial for the control of this virus, as well as improve our knowledge of its epidemiology and evolution.

## Supporting Information

S1 Fig The BI tree on the basis of E2 sequences of 37 CSFVs. Bayesian posterior probabilities above 0.80 are shown on the nodes.
(TIFF)

S2 Fig The ML tree derived from E2 sequences of 37 CSFVs. ML bootstrap values above 60% are presented on the nodes.
(TIFF)
