# Supplementary figures and images for: Time-Calibrated Phylogenomics of the Classical Swine Fever Viruses: Genome-Wide Bayesian Coalescent Approach
Source: PLoS One. 2015 Mar 27;10(3):e0121578. doi: 10.1371/journal.pone.0121578 (PMC4376735; doi:10.1371/journal.pone.0121578)

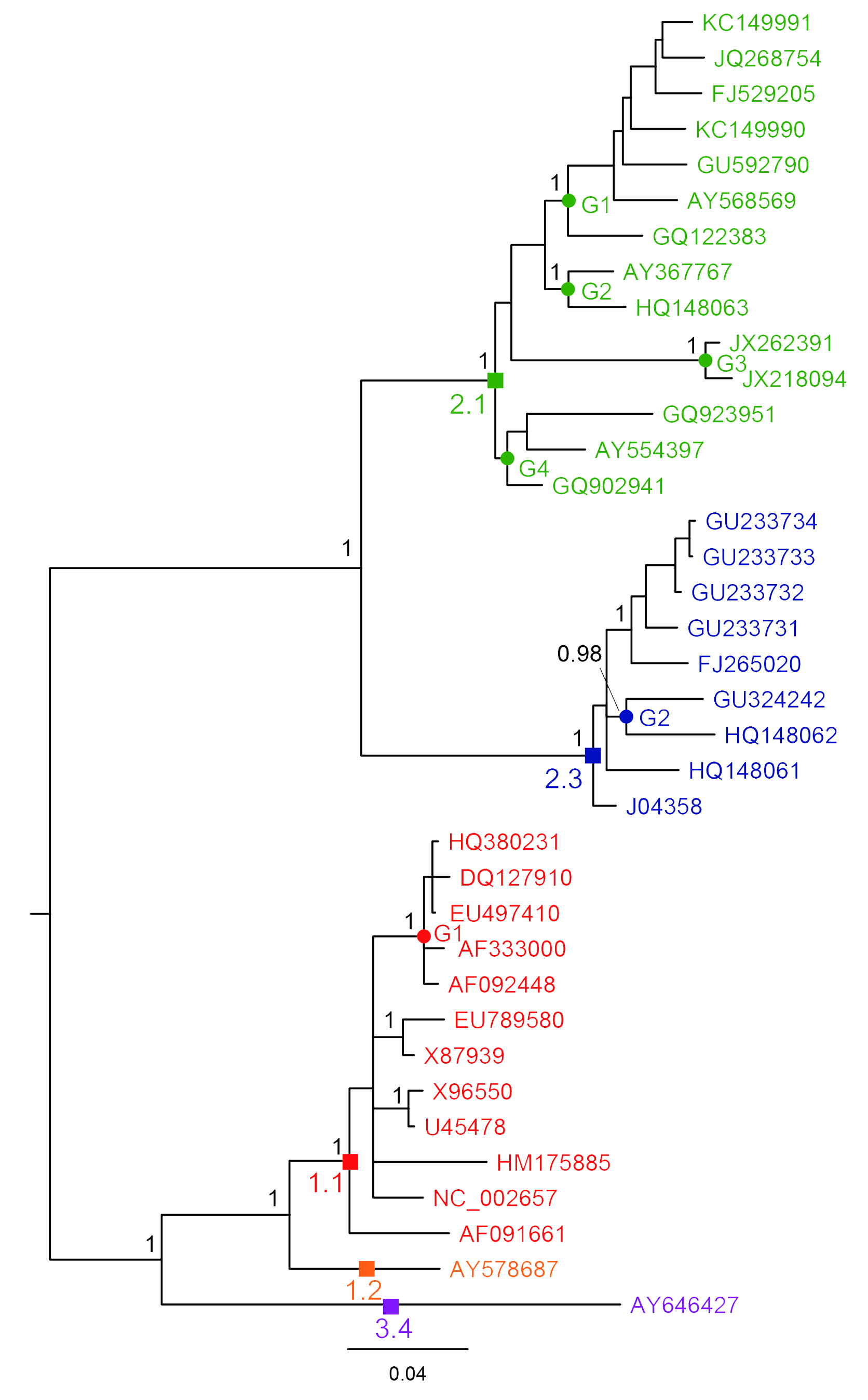

Supplement: S1 Fig — Bayesian posterior probabilities above 0.80 are shown on the nodes. (TIFF) [file pone.0121578.s001.tiff]

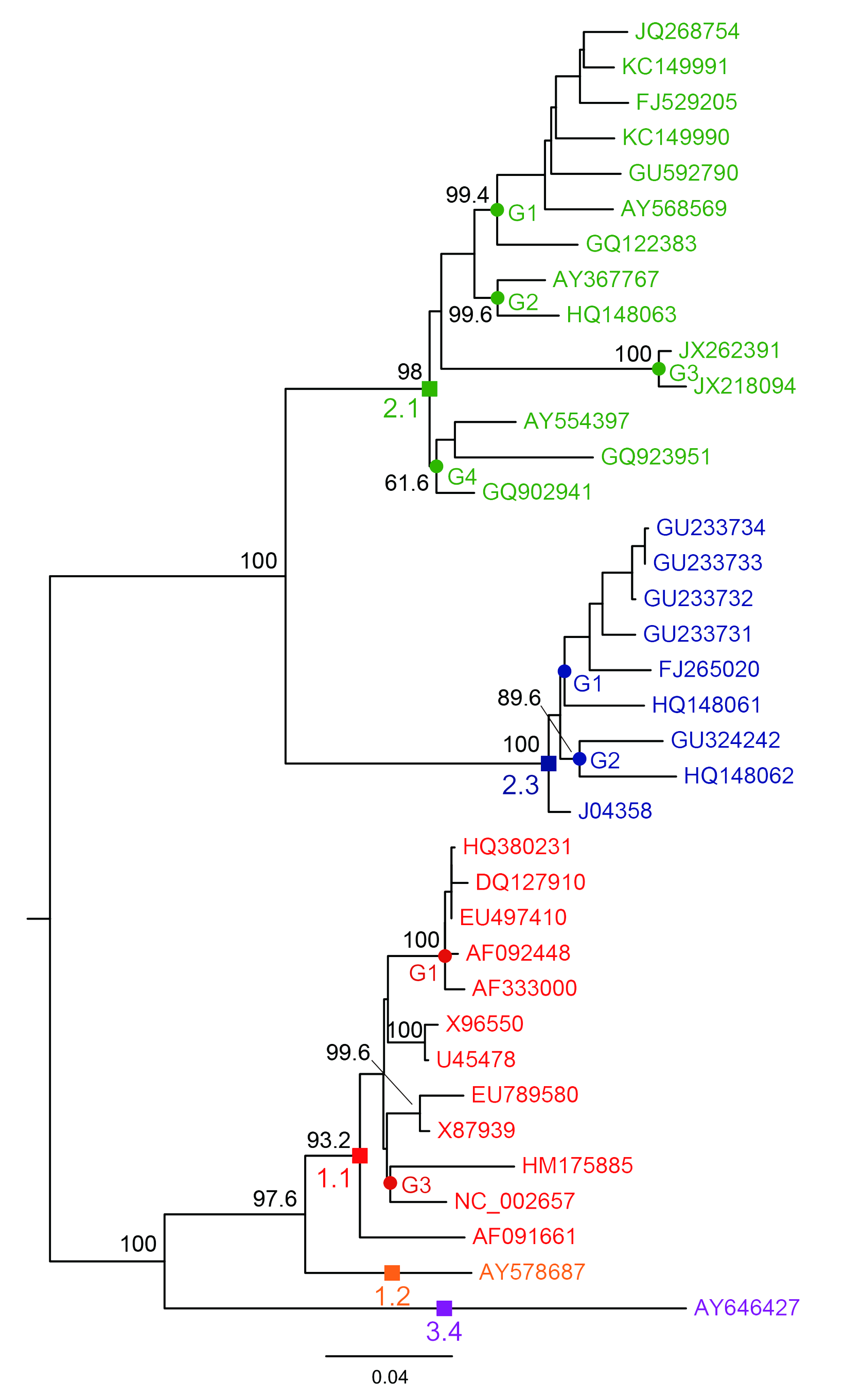

Supplement: S2 Fig — ML bootstrap values above 60% are presented on the nodes. (TIFF) [file pone.0121578.s002.tiff]
